# The Extracellular Environment of the CNS: Influence on Plasticity, Sprouting, and Axonal Regeneration after Spinal Cord Injury

**DOI:** 10.1155/2018/2952386

**Published:** 2018-04-18

**Authors:** Shmma Quraishe, Lindsey H. Forbes, Melissa R. Andrews

**Affiliations:** ^1^Biological Sciences, University of Southampton, Southampton, UK; ^2^School of Medicine, University of St Andrews, St Andrews, UK

## Abstract

The extracellular environment of the central nervous system (CNS) becomes highly structured and organized as the nervous system matures. The extracellular space of the CNS along with its subdomains plays a crucial role in the function and stability of the CNS. In this review, we have focused on two components of the neuronal extracellular environment, which are important in regulating CNS plasticity including the extracellular matrix (ECM) and myelin. The ECM consists of chondroitin sulfate proteoglycans (CSPGs) and tenascins, which are organized into unique structures called perineuronal nets (PNNs). PNNs associate with the neuronal cell body and proximal dendrites of predominantly parvalbumin-positive interneurons, forming a robust lattice-like structure. These developmentally regulated structures are maintained in the adult CNS and enhance synaptic stability. After injury, however, CSPGs and tenascins contribute to the structure of the inhibitory glial scar, which actively prevents axonal regeneration. Myelin sheaths and mature adult oligodendrocytes, despite their important role in signal conduction in mature CNS axons, contribute to the inhibitory environment existing after injury. As such, unlike the peripheral nervous system, the CNS is unable to revert to a “developmental state” to aid neuronal repair. Modulation of these external factors, however, has been shown to promote growth, regeneration, and functional plasticity after injury. This review will highlight some of the factors that contribute to or prevent plasticity, sprouting, and axonal regeneration after spinal cord injury.

## 1. Introduction

Repair of the central nervous system (CNS) after injury is one of the biggest challenges facing neuroscientists today. The most common causes of traumatic spinal cord injury (SCI) are preventable and include road traffic accidents, falls, violence, and contact sports, which often leave individuals with various types of sensory and/or motor deficits with many losing their independence. Traumatic SCI is more prevalent in males than females and occurs most commonly in young adulthood (15–29 years) and also older age (over 60) [1]. Treatment is limited for SCI and often revolves around preventing further damage with interventions involving rehabilitation being the current standard of care in the clinic [2].

### 1.1. ECM and Pathophysiology after CNS/Axonal Injury

**Figure 1:**
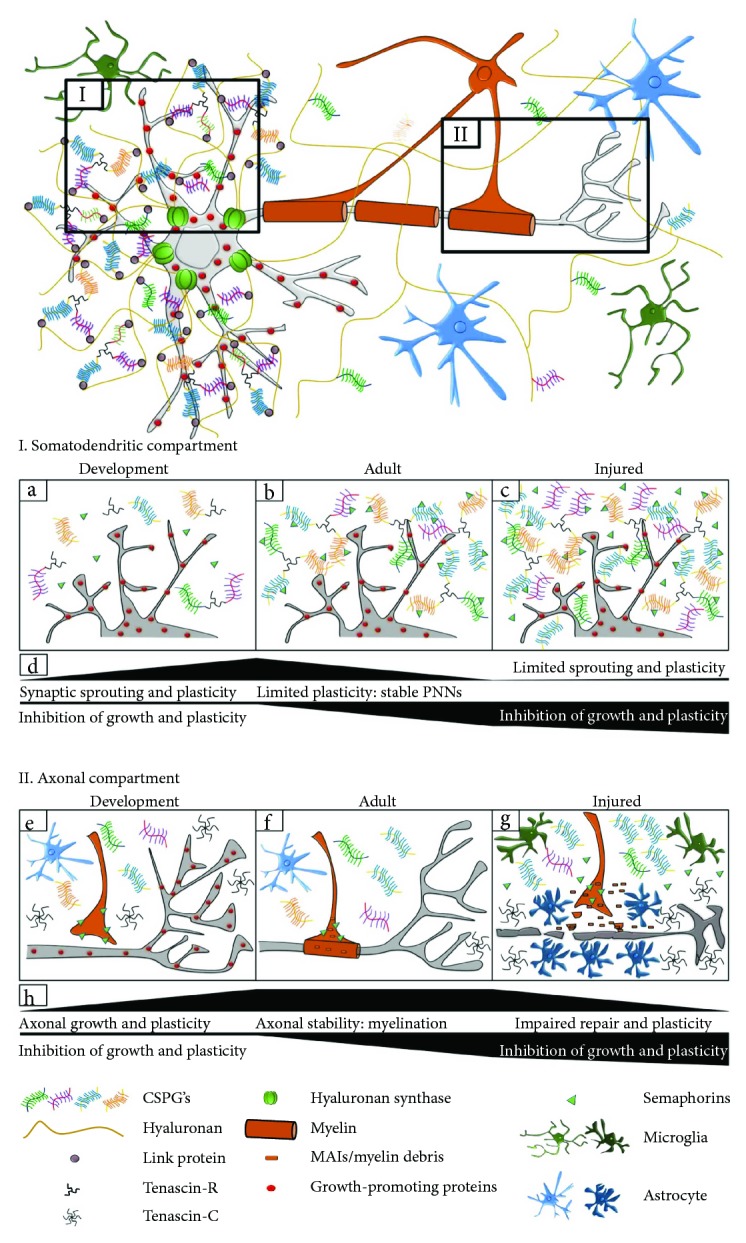
Changes in the extracellular environment during development, maturation, and injury. The extracellular environment is modified and sculpted during development in an activity- and experience-dependent manner. This gives rise to a mature and refined neuronal network in adulthood. The somatodendritic (I) and axonal (II) compartments are modified by different molecules and cells in the extracellular environment. I. (a) During development, ECM molecules such as CSPGs, tenascins, and semaphorins are upregulated. Growth-promoting factors are also expressed by neurons. These molecules aid synaptic plasticity through sprouting, growth, guidance and formation of new contacts. (b) As the CNS matures, synapses are pruned and only functionally relevant synapses are retained in adulthood. Components of the ECM, coalesce, forming PNNs around the cell body and proximal dendrites of neurons. This prevents new synapse formation and therefore limits plasticity. (c) After CNS injury, the same molecules that promoted growth during development now have inhibitory effects. CSPGs and semaphorins are upregulated, preventing growth cones forming new synaptic contacts leading to limited sprouting and plasticity. (d) The continuum of synaptic growth and plasticity increases during development but becomes limited in adulthood and further inhibited after injury. II. (e) During development, growth cones extend from unmyelinated axons to form new synaptic contacts. This is mediated by molecules that promote growth such as semaphorins, tenascins, and integrins; thus, plasticity and growth are favoured. (f) As the CNS matures, (adulthood) oligodendrocytes form mature myelin sheaths containing MAIs (Nogo-A, MAG, and OMgp), restricting aberrant sprouting. Astrocytes secrete CSPGs to limit structural plasticity. Growth-promoting proteins such as integrins and their ECM ligands (tenascins) are downregulated and absent in the axon. These factors maintain a stable environment. (g) After CNS injury, CSPGs and semaphorins are upregulated, preventing new growth cones from connecting to targets, leading to dystrophic end bulbs. Reactive astrocytes form a glial scar at the site of injury, preventing regeneration of damaged axons. Myelin debris and MAIs released from damaged myelin sheaths inhibit sprouting, axonal extension, and regeneration. Tenascin is upregulated without a concomitant upregulation of its growth-promoting integrin receptor, alpha9beta1. As such after injury, the CNS environment is not conducive to repair and regeneration. (h) The continuum of axonal growth increases during development, becomes stable in adulthood but is significantly impaired/inhibited after injury.

The CNS does not regenerate following injury due to a multitude of inhibitory factors. Intrinsically, adult central neurons are limited in their capacity to mount a regenerative response partly due to the inhibitory environment at the injury site. Several researchers have demonstrated that there is an initial growth response following injury; however, once axons encounter the inhibitory environment within the lesion, growth is arrested, leaving dystrophic axonal end bulbs in their place [3–5]. Within the normal CNS, cells are surrounded by an extracellular matrix (ECM) composed of a complex and interactive network of glycoproteins and proteoglycans [6]. Under various circumstances, these molecules can either promote neurite outgrowth such as during neuronal development [7] or repel it, such as after injury [8–20] or during disease/degenerative conditions [21] (Figure 1).

Additionally, one of the main reasons the spinal cord is resistant to repair is due to the complex and distinctive pathophysiology that occurs following injury. The underlying biology of SCI consists of a primary and a secondary phase. The primary phase is the initial injury suffered upon impact. This can include pressure on the spine, internal bleeding, as well as severed axons and nerves. Depending on the type of injury, the blood-brain barrier (BBB) may become compromised resulting in leukocyte infiltration [22, 23] as well as disruption of ionic homeostasis leading to significant changes in calcium (Ca^2+^) and potassium (K^+^) concentrations at the injury site [24, 25]. Furthermore, disruption of blood supply to the injury site can result in inadequate oxygen and nutrient supply to the affected tissue. At this stage, emergency treatment is required to prevent further damage. Emergency treatment includes stabilization of the spine often followed by surgical decompression to reduce the inflammatory response and prevent any additional bleeding. Over the subsequent weeks and months, the injury enters the secondary phase, resulting in progressive neurodegeneration. This phase is associated with a second set of symptoms including inflammation, neuronal and glial apoptosis, glutamate excitotoxicity, potential disruption of the BBB, axonal demyelination, and reactive astrogliosis, leading to the formation of a glial scar. Together with scar formation, axonal demyelination and Wallerian degeneration give rise to axonal retraction [26]. Axonal retraction occurs in two phases: (1) an early axon intrinsic, cytoskeleton-associated phase, in which Ca^2+^-dependent activation of calpain proteases leads to cytoskeletal breakdown [27] and (2) a macrophage-dependent phase, in which infiltration of phagocytic macrophages induces/correlates with retraction of dystrophic axons [28]. Alongside this, there is an upregulation of a number of inhibitory proteins including chondroitin sulfate proteoglycans (CSPGs), myelin-associated inhibitors (MAIs), and chemorepellent proteins [29]. Furthermore, the immune system reacts after injury with a concomitant and robust gliosis comprised of microglial, macrophage, and astrocyte activation and upregulation. The release of cytokines and ECM proteins associated with this gliosis may modulate the immune response and tissue damage [29]. Activation of microglia after SCI has been shown to contribute to the sustained chronic pain often suffered by individuals following injury [30, 31]. Together, this biological cascade of events not only creates an inhibitory environment but also effectively reduces axonal plasticity resulting in failed repair.

A large proportion of ongoing SCI research focuses on neuroprotection and/or treatment of these secondary mechanisms. As such, therapeutic strategies targeting removal or degradation of myelin debris and CSPGs have shown great promise for CNS repair. In this review, we will discuss some of the major contributing factors to the prevention of plasticity, sprouting, and axonal regeneration after SCI.

## 2. Myelin in the Nervous System

Myelination is an essential and unique feature of the adult nervous system providing electrical insulation and rapid propagation of action potentials over long distances, as well as metabolic support [32]. Oligodendrocytes and Schwann cells are highly specialized myelin-producing cells of the CNS and peripheral nervous system (PNS), respectively. Oligodendrocytes send out up to about 60 projections from a single cell body. Schwann cells, in comparison, form a single myelin internode around one axon. In the CNS, each oligodendrocyte projection wraps extensively around a short axonal segment to form myelin internodes [33]. This is particularly important for long axons that may be several centimeters or even meters away from the cell body. The physiological function and dynamics of oligodendrocytes and myelin have been reviewed in detail by Saab and Nave [34].

Myelination in the human brain continues over several decades with the highest volume of white matter relative to total brain volume peaking in the fifth decade of life [35]. Interestingly, higher order centres such as the cortex are myelinated later than less complex regions involved in basic neurological and homeostatic functions such as the brainstem.

Myelin is not a static structure but instead is slowly and continuously modified/remodelled over time in the healthy adult nervous system. In the visual cortex of normal aging rhesus monkeys, internodal length decreases, suggesting active remyelination occurs throughout life [36]. Indeed, shorter myelin internodes are also observed in the healthy, adult mouse during normal aging [37]. Small changes, such as internodal length, can lead to changes in conduction speed and signal timing, which is crucial for the proper function of integrated neuronal circuits [38, 39]. Just as neuronal activity associated with cognitive and motor tasks stimulates synaptic plasticity, similar activities have been reported to stimulate increases in region-dependent myelination [40]. These observations support the plastic nature of the neuroglia interaction for function.

Neuron (axon) and oligodendrocyte (myelin) cross talk is also required to maintain proper metabolic function of axons. This communication modulates many factors including localization of ion channels, cytoskeletal function, and axonal transport [41–44] and has recently been meticulously reviewed by Micu and colleagues with regard to axo-myelinic neurotransmission [45]. Myelination begins during development and as such, myelinated axons become dependent on the support provided by these glial cells [46, 47]. This dependency has been demonstrated in mice deficient in the myelin-specific proteolipid protein (PLP/DM20). Axonal swellings, associated predominantly with small-diameter myelinated axons, were observed from the age of 6 to 8 weeks in these mice [46]. Similarly, mice lacking 2′, 3′-cyclic nucleotide phosphodiesterase (CNP) in oligodendrocytes show no structural abnormality in myelin but develop axonal swellings leading to degeneration in adulthood (from 3.5 months of age and onwards) [47].

Myelination not only maximizes conduction velocity but it also controls the temporal resolution of neuronal circuits. This feature is critical for the precise control of motor, sensory, and cognitive functions. As such, neuronal circuits are able to adapt and respond to various stimuli and experiences, underpinning learning and memory. However, the adult CNS has put in place inhibitory mechanisms and molecules, such as myelin-associated inhibitors (MAIs), to prevent or minimize gross structural plasticity and remodelling that could be detrimental to the physiological function of the circuit. Although the precedence for these “protective” mechanisms is understandable for retaining a given network, it imposes restrictions on plasticity and regeneration after CNS injury. Interestingly, the situation is somewhat different in the PNS. It has long been accepted that axons within the PNS are able to regenerate after peripheral nerve damage [48]. Unlike the CNS, MAIs are either not found or are rapidly cleared by glial cells of the PNS. As such, both isolated Schwann cells and peripheral nerve grafts have been shown to create a growth-permissive environment following injury [49–51], emphasizing fundamental differences that underpin the capacity of the PNS to promote growth and repair while providing a stimulatory environment compared to the inhibitory environment of the CNS.

### 2.1. Myelin-Associated Inhibitors (MAIs)

Nogo, oligodendrocyte myelin glycoprotein (OMgp), and myelin-associated glycoprotein (MAG) have all been identified as MAIs that are able to collapse axonal growth cones and inhibit neurite outgrowth [52–56] (Figure 1) (Table 1). Nogo-A, a membrane-associated protein belonging to the reticulon family [57], is expressed predominantly in oligodendrocyte cell bodies, processes, and mature myelin. It is also expressed in several types of neurons in the developing and adult nervous systems [58]. Nogo-A was identified as a neurite growth inhibitor in the 1980's [59, 60]. This inhibition was associated with at least two distinct growth-inhibitory domains, Nogo-66 [61, 62] and Nogo-A-∆20 [63]. Evidence of the inhibitory effects of Nogo-A came from in vitro studies in which exposure of chicken retinal ganglion and rat dorsal root ganglion (DRG) neurons to Nogo-A was shown to inhibit neurite outgrowth and induce growth cone collapse [54, 64, 65]. Neutralization of Nogo-A allowed axonal growth of cocultured sensory and sympathetic neurons into optic nerve explants [60] and induced sprouting of adult rat Purkinje axons in vivo [66].

The MAI OMgp is also expressed in oligodendrocytes and in several types of CNS neurons such as pyramidal cells in the hippocampus and Purkinje cells in the cerebellum among others [67]. Although less is known about OMgp in comparison to Nogo-A and MAG, like its counterparts, it has also been shown to be a potent inhibitor of neurite outgrowth in multiple cell lines and primary neuronal cultures [56, 68, 69]. Interestingly, OMgp was originally reported to be localized to the nodes of Ranvier in the CNS and was also shown to inhibit collateral sprouting [69]. In OMgp knockout mice, impairment of CNS nodes was observed, giving rise to larger nodal lengths and greater sprouting from these nodes compared to wild-type animals, suggesting that nodal ensheathment by OMgp is important in CNS myelin development and axonal plasticity [69]. In a more recent study, however, anti-OMgp antiserum previously used to define the function of OMgp at nodes was shown to be nonspecific [70]. The antiserum displayed strong cross reactivity to the CSPG versican V2 and showed no defects in paranodes or increased nodal collateral sprouting in OMgp-null mice, potentially refuting the involvement of OMgp in node of Ranvier assembly or maintenance [70]. Despite these conflicting reports, the importance of OMgp in development and maturation of CNS myelination was shown in knockout mice that presented with hypomyelination and impaired conduction velocities in their spinal cords [71].

MAG, another MAI [52, 72, 73], is a minor component of mature, compact myelin, enriched in the periaxonal membrane of the myelin sheath and is expressed by oligodendrocytes and Schwann cells. Initially MAG was thought to promote neurite outgrowth [74]; however, in studies investigating its interaction with primary neurons, the opposite effect was observed. As such, purified recombinant MAG was found to block neurite outgrowth as well as induce growth cone retraction [52, 75]. Furthermore, Chinese hamster ovary (CHO) cells expressing MAG on their surface, cocultured with 7-day-old rat cerebellar neurons and adult DRGs, were found to inhibit neurite outgrowth [53]. In contrast, when embryonic DRG neurons were grown on MAG-CHO cells, they extended long neurites. These results highlighted the developmental regulation of neurite outgrowth and the switch to growth inhibition that occurs in adult/postnatal neurons [53]. The inhibitory properties of MAG were further confirmed by demonstrating that myelin from MAG knockout mice was not inhibitory to growth of DRG neurons in vitro as compared to myelin from normal mice [76]. Furthermore, inhibition of neurite outgrowth was completely abolished by immunodepletion of MAG from the soluble fraction of myelin-conditioned media [77]. These observations suggest that in addition to myelin debris, soluble MAIs, likely released after injury, can influence the growth capacity of neurons and axons.

### 2.2. Myelin-Associated Plasticity

In addition to the inhibitory role of MAIs on neurite outgrowth, these molecules have a significant influence in the control of plasticity in the CNS. In the developing nervous system, Nogo-A expression is high in many neurons. As such, Nogo-A has been shown to rapidly modulate actin cytoskeletal dynamics to control structural plasticity at dendritic spines of CA3 pyramidal neurons in the hippocampus in vivo [78]. Nogo-A was also shown to restrict *α*-amino-3-hydroxy-5-methyl-4-isoxazolepropionic acid (AMPA) receptor insertion, providing a mechanism by which it can regulate the strength of excitatory synaptic transmission [78]. In the cerebellum, Nogo-A expression correlates with synaptic refinement and development and has also been shown to negatively regulate dendritic morphology and synaptic transmission [79]. Neuronal Nogo-A in the adult hippocampus has also been shown to restrict physiological synaptic activity. This was demonstrated through functional antibody inhibition of Nogo-A or its receptor, Nogo-66 receptor 1 (NgR1), as well as in Nogo-A knockout mice [80]. Neutralization of Nogo-A and NgR1 resulted in increased long-term potentiation (LTP) with no change in long-term depression (LTD) in acute hippocampal slices from adult mice [80]. NgR1 expression is necessary to suppress synaptic turnover in the adult nervous system and to maintain and stabilize dendritic spines and axonal varicosities in response to Nogo-A and other ligands. In vivo time-lapse imaging in NgR1 knockout mice revealed an increase in spine dynamics without a change in total spine density in the somatosensory cortex [81].

Genetic ablation of NgR1 has also been shown to increase synaptic density in the juvenile hippocampus as well as increase spine density in primary hippocampal neurons [82]. Furthermore, in an earlier study using NgR1 knockout animals, NMDA-receptor-dependent LTD was attenuated in the Schaffer collateral (CA3-CA1) circuit of the hippocampus [83]. Conversely, in hippocampal slices of adult mice, treatment with soluble Nogo-A (Nogo66) or OMgp suppressed LTP in an NgR1-dependent manner [84]. Anti-Nogo-A or NgR1 antibody treatment of coronal brain slices from adult rat containing the primary motor cortex increased LTP through NgR1 [85]. In vivo application of the anti-Nogo-A antibody resulted in greater dendritic spine density and improved motor performance in a forelimb-reaching task compared to control antibody-treated animals [85]. Importantly, anti-NogoA antibody treatment also demonstrated that in an uninjured system, inhibition of the Nogo pathway induced sprouting of CA3 fibers [86]. In a study by Zemmar and colleagues, mouse lines in which Nogo-A was knocked out in either oligodendrocytes or neurons revealed enhanced dendritic branching and spine formation, suggesting that both sources of Nogo-A contribute to synaptic development and plasticity [87].

Recently, the G-protein-coupled receptor for Nogo-A-Δ20, sphingolipid 1-phosphate receptor 2 (S1PR2), was discovered. It was shown that Nogo-A-Δ20 binds S1PR2 to mediate its growth-inhibitory effects by signaling through the G protein G_13_, leukemia-associated Rho guanine exchange factor (LARG) and RhoA to mediate actin destabilization and growth cone collapse [88]. Furthermore, similar to Nogo-A neutralization strategies using antibodies or knockdown paradigms, inhibition of S1PR2 blocked Nogo-A-Δ20, thus counteracting Nogo-A-Δ20's inhibition of neurite outgrowth and cell spreading. S1PR2 inhibition also increased hippocampal and cortical LTP, thus modulating synaptic plasticity [88]. These results demonstrate that MAIs and their receptors are able to antagonize growth-promoting pathways to modulate synaptic strength and transmission influencing synaptic plasticity.

Nogo-A and NgR1 have also been shown to be important for regulating memory formation and regulating the acquisition of different behavioural tasks [81, 85, 89, 90]. For example, Karlsson and colleagues demonstrated impaired locomotor function and novel object recognition in mice lacking NgR1 [90]. Overexpression of NgR1 impaired spatial learning memory with a reduction in mature spine density and complexity [90]. Furthermore, the Morris water maze test of spatial learning also revealed Nogo-A knockout mice were able to find the hidden platform faster than wild-type animals and had better long-term memory retention. This improvement in learning performance correlated with increased dendritic spine density in CA3 apical dendrites [91].

The involvement of MAIs in limiting neuronal plasticity has also been elegantly demonstrated in the rodent visual system. In the juvenile brain, critical periods (CP) of experience-driven plasticity occur. These are important for the normal development of the primary visual cortex. As such, visual experiences during the CP refine the neuronal network of the visual system enabling the formation of ocular dominance columns in the cortex. At the end of the CP, mature networks that have been formed are actively maintained, thus restricting plasticity [92]. In NgR1 and Nogo-A mutant mice, although plasticity occurs during the CP, it has also been shown to continue beyond the normal length of the CP [93, 94]. As a consequence, robust ocular dominance plasticity associated with monocular deprivation persists throughout adulthood [93, 94]. Similarly, ocular dominance plasticity was extended in the mouse knockout of the MAI receptor known as paired immunoglobulin-like receptor B (PirB) [95]. These studies demonstrate that MAIs and their receptors stabilize the neuronal circuit and limit experience-driven neuronal plasticity. Thus, MAIs like many other proteins are involved in consolidation and closure of CPs through stabilization of mature, highly tuned, and elaborate neuronal networks.

### 2.3. Combatting Myelin Inhibition to Promote Neurite Outgrowth and Axonal Regeneration

As discussed, myelin contains several growth-inhibitory molecules that limit both plasticity and axonal regeneration in the injured mammalian CNS (Table 1), several of which have been extensively reviewed elsewhere [34, 96, 97]. A number of strategies to counteract and neutralize the effects of MAIs starting with complete demyelination have shown improvements for axonal regeneration and functional recovery. Studies from Keirstead and colleagues in the 1990's showed that following complete or partial demyelination, in and proximal to the lesion site, promoted axonal regeneration with extensive neurite outgrowth [98, 99]. Specifically, after a lateral hemisection of the adult rat spinal cord, transient disruption of mature myelin by intraspinal fusion of serum complement proteins and a myelin-specific antibody (anti-galactocerebroside, the major sphingolipid in myelin) was shown to facilitate axonal regeneration of rubrospinal axons into the caudal lumbar spinal cord [98]. Using a similar anti-myelin approach, Keirstead and colleagues demonstrated that demyelination induced by antigalactocerebroside and complement, combined with Schwann cell transplantation, enhanced axonal regeneration following injury [99]. Similarly, in studies aimed at blocking Nogo-A activity with anti-Nogo-A antibodies, there was significantly increased regeneration and sprouting of transected corticospinal tract (CST) axons in rats after spinal cord lesion compared to controls [100, 101]. Additionally, anti-Nogo-A treatment promoted compensatory fiber sprouting and innervation from the uninjured side to the denervated side [100].

In studies targeting MAI receptors, there have been several promising studies enhancing neurite outgrowth and axonal regeneration upon modulation of these receptors. The NgR1 receptor lacks a transmembrane domain and therefore forms a complex with other transmembrane co-receptors such as LINGO-1 (LRR and Ig-containing Nogo receptor-interacting protein) and p75NTR (p75 neutrotrophin receptor) or TROY (tumor necrosis factor receptor superfamily member). In doing so, it can transduce the myelin inhibitory signal to the axon, thus mediating growth cone collapse and growth arrest of neurites [102]. Remarkably, all three MAIs bind to and activate the same NgR1 receptor complex [61, 68, 103]. All three MAIs also bind to the PirB receptor [103]. Blocking either NgR1 or the PirB receptor partially attenuates growth cone collapse and neurite outgrowth in DRG and cerebellar granule neurons in the presence of MAIs or myelin. However, in NgR1 null cerebellar neurons, antibody-mediated PirB inhibition suppressed the inhibitory effects of MAIs on neurite outgrowth [103]. In vivo, PirB is reported to play a key role in cortical plasticity [95]. In contrast to in vitro observations, CST regeneration was not enhanced in PirB-knockout mice after SCI [104]. A number of other genetic deletion/mutation studies of MAIs and their receptors have provided further evidence regarding the inhibitory action of these proteins on growth. However, effects after injury in NgR1 or Nogo-A, MAG and OMgp knockout mice have been variable [105–107]. In triple mutant mice (Nogo-A, MAG, and OMgp), greater axonal growth and sprouting of CST and raphespinal tract axons and improved locomotion were observed after SCI compared to single or double knockouts [108]. However, Lee and colleagues observed limited axonal sprouting and regeneration of CST axons after SCI with no synergistic effects in triple knockout animals [107]. This variability may likely be due to the location and type of injury as well as compensation and synergism between MAIs and other growth-inhibitory factors in single knockout animals. Indeed, growth-inhibitory molecules such as EphrinA3, signaling via the EphrinA4 receptor, are upregulated in the spinal cord of Nogo-A knockout mice, thus suggesting that compensatory mechanisms mediate the residual inhibition observed in these animals to restrict neurite outgrowth and repair [109].

Combatting myelin inhibition with other treatment strategies including vaccines, monoclonal antibodies (including the anti-Nogo A antibody), and peptide ligands to compete for receptor binding [62, 110] have shown significant potential for CNS repair [111]. After either acute or chronic SCI in rodents, treatment with a soluble NgR1 decoy protein (NgR1(310) ecto-Fc) that neutralizes the effects of all three MAIs (Nogo-A, MAG, and OMgp) has shown promising results in promoting axonal sprouting and functional recovery [112–114]. Interestingly, after a spinal dorsal column crush injury in adult rats, inclusion of either a preconditioning peripheral sciatic nerve lesion and application of NgR1 decoy protein treatment or Chondroitinase ABC (ChABC—an enzyme which digests the glycosaminoglycan sugar chains of CSPGs) treatment led to greater axonal regeneration [115]. Combining two of these treatments did not enhance regeneration any further; however, combining all three promoted axons to regenerate millimeters past the lesion site [115].

Acute antibody blockade of Nogo-A alone has been shown to enhance axon sprouting in the injured spinal cord as well as improve behavioural outcomes [100, 101, 116]. In a rat model of stroke, anti-Nogo-A antibody treatment increased dendritic arborization and spine density [117]. Similar treatments have also resulted in increased sprouting and fiber regeneration after lesion in rat organotypic hippocampal slice cultures [86]. Antibodies against Nogo-A delivered via injection of hybridoma cells producing IN-1 antibody (raised against an inhibitory fraction of myelin) into the frontoparietal cortex 7–10 days prior to the spinal cord lesion promoted regeneration of axons up to 7–11 mm in transected CST axons within 2–3 weeks postinjury [116]. In a complimentary study, adult rats implanted with IN-1-secreting hybridomas in the hippocampus immediately after dorsal overhemisection of the spinal cord showed greater improvements in locomotor function [101]. A study using intrathecal administration of anti-Nogo-A antibodies into the subdural space also induced regeneration of CST axons alongside behavioural improvements in open field locomotion, ladder walking, narrow beam walking, as well as improvements in response to heat and von Frey hairs [100]. Due to the successful regeneration observed in experimental injury models treated with anti-Nogo-A antibodies as described above, clinical trials are now underway using these antibodies for the treatment of stroke and SCI [111, 118].

### 2.4. Remyelination after Injury

Despite the multitude of inhibitory factors associated with myelin, replacement of oligodendrocytes and myelin around demyelinated axons is thought to be a key mechanism in promoting CNS regeneration, with the aim of reinstating neuron-glia cross talk and functional recovery. Mature oligodendrocytes, like neurons, are postmitotic cells and therefore do not contribute to remyelination after injury. Remyelination is established predominately by proliferating oligodendrocyte precursor cells (OPCs) expressing platelet-derived growth factor receptor A, neuron-glial antigen 2 (NG2) cells, but also neural stem and progenitor cells (NSPCs). These cells migrate to the lesion site where they differentiate and give rise to adult-born, myelinating oligodendrocytes following injury [119–122]. This robust oligodendrogenesis occurs throughout the first few months after injury, which partially compensates for loss of mature oligodendrocyte and myelin [123]. Newly formed oligodendrocytes have been shown to generate myelin sheaths in a short space of time [124]. As such, plastic changes associated with de- and remyelination are possible. Indeed, genetic ablation of new myelin-forming oligodendrocytes impairs performance on a complex wheel motor task in mice [125].

MAIs and other growth-limiting proteins are important for maintaining physiological plasticity and neuronal network stability. Unfortunately, this effectively restricts/prevents surviving neurons/networks from re-establishing functional connectivity after injury or disease. The importance of myelination for the function of the CNS has been described earlier and it is therefore understandable that after injury, where axonal degeneration and demyelination occur, spontaneous remyelination also occurs to stabilize vulnerable axons [126, 127]. However, similar to the inadequate axonal regeneration that occurs following injury, the extent and quality of remyelination is also thought to be inadequate, resulting in incomplete, abnormally thin myelin sheaths and continued axonal dysfunction [128, 129].

Many studies have reported a high prevalence of very thin myelin surrounding lesion zones even years after SCI. In adult rats, incomplete myelination was reported up to 450 days following contusive SCI [130]. In postmortem chronically injured human spinal cords, 1–22 years following injury, immunohistochemical analysis also demonstrated some axonal demyelination even a decade following SCI [131].

Experimentally, after a chronic contusive SCI, the majority of spared rubrospinal axons are demyelinated in rats [132] and mice [133]. By measuring spared rubrospinal axons that traverse the injury site with shortened internodal lengths (indicative of remyelination), it was found that the majority of spared axons were remyelinated by 3 months after SCI [132]. Hesp and colleagues also demonstrated remyelination of spared axons 2–3 months after contusive SCI, albeit stereotypically shorter and thinner, suggesting remyelination occurs continuously and chronically after SCI [123]. In a subsequent study, Powers and colleagues measured spontaneously regenerated myelin in vivo [120]. Consistent with previous studies, they found that early after injury, the mean length of regenerated myelin sheaths was significantly shorter than in uninjured myelin; however, by 6 months post-injury, the newly formed myelin sheaths and internodes were similar to controls [120]. These studies suggest that abnormally thin and shorter myelin internodes observed many months post-injury/pathology may be due to ongoing demyelination and not incomplete myelin regeneration to intact spared axons.

Spontaneous remyelination after SCI is slower and less efficient than remyelination following chemically-induced focal demyelination. In mice, after lysolecithin-mediated focal demyelination, myelin debris is rapidly cleared within the first 4 days [134], remyelination is observed at 7 days and is complete by 3 weeks [135]. The rate of remyelination after SCI is likely due to the inhibitory environment and resultant pathology including myelin debris and associated MAIs (NogoA, MAG, and OMgp) which are themselves inhibitory to OPC differentiation [136, 137]. It is clear that the interplay of demyelination and compensatory remyelination postinjury is important for functional recovery. Several sources of myelinating cells have been examined as transplantation-based therapies for SCI [4, 138, 139]. Such treatments have led to improvements in behavioural recovery after contusion SCI [140].

Like OPCs, neural progenitor cells also become activated and migrate to the site of injury. However, they predominantly differentiate into reactive astrocytes [121, 122, 129]. This migration of cells contributes to the inhibitory environment produced by MAIs in and around the glial scar following SCI, thus precluding axonal regeneration [141]. In addition to targeting MAIs as a barrier to neuroregeneration after injury, other molecules such as components of the ECM, which work synergistically to maintain a stable, predominantly growth-inhibitory environment also need to be considered and are discussed in subsequent sections.

## 3. CSPGs and Perineuronal Nets

The ECM of the CNS is rich in CSPGs, some existing within the extracellular milieu and others associated with specific structures. Within the CNS, CSPGs can associate into specialized structures termed perineuronal nets (PNNs) which surround the soma and dendrites of mature neurons. PNNs are comprised of ECM proteins including hyaluronan, linking proteins [e.g., cartilage link protein 1 (Crtl1/Hapln1) and brain-specific link protein (Bral2/Hapln4)], CSPGs, and tenascin-R (TN-R) [142], with one further study also identifying tenascin-C (TN-C) by immunoblot within PNNs [143] (Figure 1) (Table 1). There are also a number of CSPGs, including brevican, neurocan, aggrecan, phosphacan, and versican, which bind to the hyaluronan backbone of the PNN (Table 1) [144]. Hyaluronan is synthesized by hyaluronan synthases (HAS). Its interaction with CSPGs occurs via link proteins (e.g., Hapln1). TN-R molecules function to cross-link CSPGs to form an organized PNN backbone. Additionally, TN-R has been shown to interact with integrins, such as *β*1 integrins, contactin/F3/F11, phosphacan, and MAG [145–147]. Recently, semaphorin 3A (sema3A) and 3B (sema3B) (discussed below) have also been found to be associated with the CSPGs within the PNN structure [148].

Although PNNs have been localized to some glutamatergic neurons in the CNS [149], they predominantly associate with the neuronal cell body and proximal dendrites of fast-spiking parvalbumin-positive GABAergic interneurons, forming a robust lattice-like structure [144, 150]. Maintenance of this specialized structure is important for synaptic and network stabilization and homeostasis. Specifically, PNNs function to stabilize mature neurons by reducing dendritic spine plasticity [151], forming a scaffold for synaptic inhibitory molecules [152], and also restricting the movement of receptors at the synapse [153]. Formation and maturation of PNNs is concurrent with the development and maturation of the nervous system during CPs. Enzymatic digestion of this specialized ECM structure with ChABC prolongs the CP in the visual system [154]. When specific components of PNNs are deleted, such as link protein 1 (Crtl1), PNN formation is reduced and neuronal plasticity is enhanced [155]. Furthermore, primary neuronal and astrocytic cultures from quadruple knockout mice that lack the CSPGs neurocan, brevican, as well as TN-C and TN-R have reduced PNN formation. In these cases, the mice display changes in synaptic plasticity, impaired synaptogenesis, synapse stability, and altered synaptic activity [156].

### 3.1. The Glial Scar and Associated Inhibitory Proteins

As discussed above, unlike the PNS, the adult mammalian CNS has a reduced ability to regenerate. This is partly as a result of the formation of a characteristic glial scar created at the injury site which surrounds the lesion with reactive astrocytes and fibroblasts and inhibits axonal regeneration [29]. Furthermore, it has been shown by Davies and colleagues that when glial scarring is minimized, robust CNS regeneration can occur even in areas of degenerated white matter in the spinal cord [157, 158].

CSPGs are the main family of inhibitory proteins associated with the glial scar and axonal injury [8–10]. These include the subgroup lecticans (comprising aggrecan, brevican, versican, and neurocan), small leucine-rich proteoglycans (including biglycan and decorin), phosphacan/receptor-type-protein-tyrosine phosphatase *β* (RPTP*β*), and other proteoglycans, including NG2 and neuroglycan-C [159]. After injury, CSPGs are actively secreted into the ECM, mainly by reactive astrocytes and neurons [160], but with a minor component also coming from secretion by macrophages and oligodendrocytes [145, 161, 162]. This leads to an abundance of CSPGs at the injury site, adding to the inhibitory milieu. The main feature contributing to the inhibitory nature of CSPGs is their structure as they have a number of glycosaminoglycan (GAG) side chains. Each CSPG varies on the number of side chains they have; for example, aggrecan contains over 100 GAG side chains while others contain less than 10 [159]. The number of chains and the sulfation status of CSPGs equates to the functional effect of the CSPGs on CNS growth [10, 163–165].

CSPGs impose inhibitory effects on both oligodendrocytes and neurons. This has been shown to be mediated through the protein tyrosine phosphatase sigma (PTP*σ*) receptor. When CSPGs bind PTP*σ* receptors, they initiate activation of the GTPase Rho/ROCK signaling pathway. In neurons, this results in an inhibition of axonal growth, converting growth cones into a dystrophic state [13–15]. Using a novel peptide mimetic of PTP*σ*, Lang and colleagues were able to block CSPG-mediated inhibition in vitro, enabling adult sensory neurons to extend axons through a CSPG gradient [15]. This was equivalent to ChABC treatment, which removes the GAG sugar chains containing the PTP*σ* ligand. Administration of the PTP*σ* peptide mimetic after contusive SCI also facilitated functional recovery of both locomotor and urinary systems, suggesting reconnection of functional circuits [15]. When the PTP*σ* receptor is not present, animal models of SCI show increased regeneration of CST axons [166]. This was evidenced through RNAi-mediated downregulation of PTP*σ*, which negated the inhibitory effects of CSPGs on oligodendrocyte process outgrowth and myelination. The same effect was observed in oligodendrocytes isolated from PTP*σ* knockout mice [167]. Together, these results highlight the inhibitory role of CSPGs on OPC migration, maturation, and myelination, limiting their reparative potential after injury.

Interestingly and contrary to the widely accepted demonstration of glial scar-mediated inhibition of axon regeneration, recent evidence by the Sofroniew lab has shown that the glial scar has a beneficial effect for regrowth and not only acts a barrier to prevent further damage but also may be advantageous or at least necessary for the repair process. Specifically, in a 2016 study by Anderson and colleagues examining spinal cord repair in two different transgenic mouse models with reduced glial scar formation either via deletion of scar-forming astrocytes (TK+GCV; thymidine kinase + ganciclovir) or via conditional knockout of STAT3 (signal transducer and activator of transcription 3) specifically in astrocytes, they did not find an increase in regeneration, and in some axonal tracts (CST and ascending sensory tracts), there was an increase in axonal dieback after injury [168]. Despite acting as a physical barrier to axonal regrowth, results from this study suggests that the glial scar modulates the inflammatory response after injury and that without the glial scar present, this inflammatory response can be an equivalent barrier to regrowth [168]. Nevertheless, a recent study characterizing the varying phenotypes of astrocytes specifically in regard to a lesion site was published in 2017 by Hara and coworkers [169]. In this study, three distinct subtypes of astrocytes associated with the glial scar were characterized: the naïve astrocytes present prior to lesion, the reactive astrocytes which are first to infiltrate the lesion milieu and are classified as “transitional” with potential neuroprotective and immune-regulatory roles, and the scar-forming astrocytes which comprise the late stage growth-inhibitory astrocytic scar [169]. Intriguingly, when the “transitional” (reactive) astrocytes were transplanted into naïve spinal cord, they reverted to naïve astrocytes; likewise, they converted to scar-forming astrocytes when transplanted into an injury site, demonstrating that the environment dictates astrocytic phenotype and consequently glial scar-mediated inhibition [169]. This phenotypic change was found to largely be controlled by collagen type I found in high levels within the lesion and shown to be integrin-N-cadherin-dependent [169]. Without intervention, the normal fate of astrocytes in close proximity to a CNS lesion is directed to the scar-forming astrocytic phenotype, which indeed is detrimental to axonal regeneration. A recent commentary by Silver on glial scar-mediated inhibition and the inability for mammalian lesions to form growth-promoting astroglial bridges or to otherwise enhance glial-mediated regrowth articulates this issue and further elucidates the need for treatments to counteract the glial scar [170] such as with ChABC or others.

Enzymatic inactivation of CSPGs by ChABC renders them unable to interact with their receptors. This has been shown to be effective in promoting axonal plasticity and regeneration in many models of SCI. Specifically, following a cervical dorsal column lesion and treatment with ChABC, dorsal column and CST fiber growth extended beyond the injury compared to control, untreated groups where the axons retracted away from the lesion [171]. In this study, ChABC also promoted sensory and proprioceptive behavioural recovery after injury [171]. Furthermore, in studies combining ChABC treatment with forelimb rehabilitation after dorsal column crush lesion, significant axonal regeneration and behavioural recovery also resulted when treated at the time of injury [172] or with delayed treatment 4 weeks post-injury [113]. In a rat model of compressive SCI, treatment with ChABC promoted oligodendrocyte differentiation and myelination of endogenous and transplanted neural precursor cells [173, 174]. ChABC treatment also promoted migration of OPCs in and around the lesion site following a spinal contusion injury [175]. The inhibitory activities of CSPGs on OPC differentiation and myelination have also been counteracted with small molecules such as protamine, a heparin antagonist [176].

The presence of CSPGs in and around the lesion site after SCI as well as in multiple sclerosis has been found to negatively correlate with successful remyelination. In vivo and in vitro observations show that CSPGs limit migration and maturation of OPCs. In models of demyelination, lesion-induced CSPG upregulation reduced differentiation and process development of OPCs. Clearance of CSPGs by xyloside treatment after lysolecithin-induced demyelination not only increased the number of OPCs and oligodendrocytes present in the lesion site but also significantly improved remyelination [177]. Upregulation of NG2, a CSPG found on the surface of OPCs, was also observed after lysolecithin-induced demyelination. Remyelination and repair only occurred after removal of NG2 deposits from the lesion site, mediated by matrix metalloproteinase-9 [178].

Because ChABC is an enzyme, it has its limitations; primarily degradation over time [163] and therefore requires repeated dosing to maintain adequate enzymatic activity to promote repair. In response to this requirement, long-term expression of ChABC has been achieved in rat models of SCI using lentivirus or adeno-associated virus [179], promoting regeneration of CST axons [180]. It is unclear, however, whether constitutive expression of ChABC over a long period of time may result in unwanted plasticity within the CNS.

### 3.2. Tenascin-C-Mediated Inhibition of CNS Plasticity and Axonal Regeneration

Alongside increased levels of CSPGs, there are a number of developmental proteins that are upregulated after axonal injury in the mature CNS, including tenascins, semaphorins, ephrins, and netrins [181]. In this review, we will discuss the role of tenascins and semaphorins in axonal growth and regeneration. Tenascins are extracellular glycoproteins that form a constituent part of the ECM [182]. TN-C is the most abundant glycoprotein within the CNS ECM, which is highly expressed during development but is downregulated as the mammalian CNS matures. TN-C has vastly opposing roles in both the developing and mature CNS, acting as a growth-promoting molecule in certain circumstances and a growth-inhibitory molecule in others. For example, in the developing CNS, these proteins, specifically TN-C, promote neural precursor proliferation and migration along with axonal extension, guidance, and growth cone formation [7]. On the other hand, the pattern of TN-C immunostaining in the developing CNS demonstrates that it can also be inhibitory to growth as it contributes to the formation of boundaries between neuroanatomical subdivisions, showing its chemorepulsive role [183].

Upon injury in the mature CNS, TN-C is upregulated and deposited in the ECM by reactive astrocytes, contributing to the inhibitory glial scar that forms after SCI (Table 1) [11, 12, 16–20]. Conversely, levels of TN-R are increased as the mammalian CNS matures due to the increased formation of PNNs as discussed above [184]. Generally, TN-C exerts an inhibitory effect on mature axons; however, when bound to a specific heterodimeric integrin receptor, alpha9 beta1 integrin (*α*9*β*1), which is also downregulated in the mature CNS, TN-C can promote neurite outgrowth and axon regeneration [17, 19]. Unfortunately in the mature CNS, a concomitant upregulation of its integrin receptor *α*9*β*1 does not occur. Integrins, such as the *α*9*β*1 heterodimer, are highly expressed during CNS development at the axonal cell surface to aid growth cone formation and axonal elongation. The imbalanced upregulation of TN-C after injury without expression of its growth-promoting receptor has been shown to substantially contribute to the intrinsic failure of axonal regeneration. Similar to the situation during CNS development, TN-C (specifically the fibronectin III binding domain, fnD) has also an opposing role in that it has been shown to be necessary for regeneration [18]. Specifically, spinal cord compression lesion in adult mice led to more pronounced dying back of severed CST axons in TN-C knockout mice, which was rescued upon replacement of TN-C via viral-mediated overexpression of fnD [18].

Both the growth-promoting and inhibitory effects of TN-C and TN-R have been elucidated in a number of studies. Interestingly, TN-C and TN-R exert opposite functions in facial nerve injury. Specifically, recovery of vibrissal movement was worse in TN-C deficient mice and better in TN-R deficient mice compared to controls [185]. Single knockout studies of TN-R and TN-C show impaired LTP and plasticity in vivo and in vitro [186, 187]. TN-C deficient animals subjected to hemisection in the lumbar spinal cord showed increased numbers of neurofilament-positive fibers along the hemisected edges of the spinal cord [188]. In contrast, deficiency of TN-R improves functional recovery of mice after compression of the thoracic spinal cord [189] with further studies using antibodies against TN-R showing improved recovery following SCI [190].

### 3.3. Semaphorin-Mediated Inhibition of CNS Plasticity and Axonal Regeneration

Semaphorins are a class of membrane-associated proteins involved in axonal growth, guidance, and formation of new synaptic connections during embryonic development [191]. Semaphorins are also expressed within the adult CNS. In addition, studies of PNNs have demonstrated that the semaphorins sema3A and 3B are associated with the CSPGs in PNN structures suggesting that these semaphorins contribute inhibition and/or chemorepulsion of axon growth in part by their contribution to PNNs [148]. Specifically, sema3A was localized to *Wisteria floribunda agglutinin*- (WFA-) positive PNNs, an association that was eliminated following treatment with ChABC [148]. Semaphorins have also been detected within the glial scar post-injury (Table 1) [192–194]. Specifically, Sema3A is found to be upregulated after CNS injury [192] and has likewise been linked to the prevention of regeneration after SCI [195, 196]. In addition, an inhibitor of sema3A, SM-216289, has been shown to promote axonal regeneration, enhance Schwann cell migration to the lesion site, inhibit apoptosis, and significantly improve functional recovery of the hindlimb following transection of the spinal cord in adult rats [195]. Interestingly, particular semaphorin subtypes, such as sema4D, have also been found within the membrane of myelinating oligodendrocytes [197]. Furthermore, after CNS injury, sema4D is upregulated and can prevent axonal growth in vitro, suggesting it may contribute to the myelin-associated inhibitory environment at the lesion site in vivo [197].

## 4. Summary

Understanding the physiological regulation of axonal growth and plasticity in the adult nervous system is essential to the development of therapeutic strategies. Plasticity and regeneration in the CNS, or lack thereof, are heavily regulated by ECM formation. As the CNS matures, giving rise to myelinated axons, PNNs, and associated proteins, it is reasonable to understand why there is a reduced capacity for plastic changes and axonal repair (summarized in Figure 1). These extraneuronal components are key to maintaining the circuitry required for normal nervous system function both at the level of the cell soma and dendrites as well as the axons. As such, the CNS has protective measures in place to prevent or limit new axonal growth and/or connections so that mature networks are not compromised. It is therefore detrimental for the CNS to allow axonal growth and plasticity (at a gross level) to continue throughout adulthood, outside of normal synaptic plasticity. It is clear from the literature, however, that the CNS is capable of mounting a regenerative response if inhibitory factors are (a) removed, creating a growth-permissive environment or (b) temporally regulated providing a sufficient window for regeneration to occur. By continuing to understand changes that promote plasticity and aid repair in the PNS as well as those that prevent repair in the CNS, we can therefore assess key targets for therapeutic intervention. Going forward, it is highly unlikely that a single target therapeutic strategy will be successful in fully promoting both axonal regeneration and behavioural/functional recovery after SCI. Approaches which confront issues with both the extracellular environment of the CNS together with the internal environment of the neuron have the best opportunity for achieving successful repair.

## Figures and Tables

**Table 1:** CNS Inhibitors from myelin, proteoglycans, and ECM. This table summarizes the main inhibitors in the CNS originating from myelin, proteoglycans, and ECM. The table includes some of the inhibitory effects of these molecules in the CNS as well as experimental interventions utilized and trialed to counteract the inhibition. This table is by all means not exhaustive but does highlight several influential studies that have demonstrated inhibition in the CNS and/or ways to counteract it.

**Molecule**	**Inhibitory effect**	**Interventions to counteract inhibition**	**References**
*Myelin-associated inhibitors (MAIs)*			

Myelin-associated glycoprotein (MAG)	Inhibits neurite outgrowth	MAG KO mice	[52, 53, 72–77, 107, 108]
	Induces growth cone retraction	Immunodepletion of MAG	

Nogo-A	Inhibits neurite outgrowth; induces growth cone collapse	Anti-Nogo A antibody	[54, 55, 57–66, 78–80, 84–87]
	Restriction of synaptic plasticity and learning/memory		[91, 93, 94, 100, 101, 107, 108, 116–118]

Oligodendrocyte myelin glycoprotein (OMgp)	Inhibits neurite outgrowth	OMgp KO mice	[56, 67–71, 84, 107, 108]
	Inhibits collateral sprouting		

*MAI Receptors*			

Nogo-66 receptor 1 (NgR1)	Inhibits neurite outgrowth; induces growth cone collapse	NgR1 KO mice	[61, 62, 68, 80–85, 89, 90, 93, 94]
	Restriction of synaptic plasticity and learning/memory	Blocking NgR1 receptor	[102, 103, 105, 106, 112–114]

Paired immunoglobulin-like receptor B (PirB)	Restriction of ocular dominance plasticity; inhibits neurite	PirB KO mice	[95, 103, 104]
	Outgrowth; induces growth cone collapse	Blocking PirB receptor	

Sphingolipid 1-phopsphate receptor 2 (S1PR2)	Inhibits neurite outgrowth; restriction of synaptic plasticity	Blocking S1PR2 receptor	[88]

*ECM and glycoproteins*			

Chondroitin sulfate proteoglycans (CSPGs—*brevican*, *neurocan*, *aggrecan*, *phosphacan*, *versican*)	Localized to PNNs; restriction of plasticity through synaptic	ChABC enzyme - removes CS-GAGs of PNNs	[10, 13–15, 113, 142–144, 155]
	Stabilization in mature neurons;	Novel peptide-mimetic of PTP*σ* receptor	[159–163, 168–177]
	Secreted by reactive astrocytes of glial scar limits remyelination	Xyloside treatment (after demyelination)	

Link proteins (*Crtl1/Hapln1*; *Bral2/Hapln4*)	Localized to PNNs; restriction of plasticity through synaptic stabilization in mature neurons	Crtl1 conditional KO mice (neurons)	[142, 143, 154]

Hyaluronan	Localized to PNNs; restriction of plasticity through synaptic stabilization in mature neurons	Inhibitor not yet tested in CNS	[142, 143]

Tenascin-R (TN-R)	Localized to PNNs; restriction of plasticity through synaptic stabilization in mature neurons;	TN-R KO mice	[7, 16, 142, 144–146, 155, 182, 183, 186, 187]

Tenascin-C (TN-C)	Creates neuroanatomical boundaries for growth in development secreted by reactive astrocytes after injury	TN-C KO mice	[7, 17–19, 155, 179, 180, 183–185]
		Reexpression of *α*9*β*1 receptor	

Semaphorins	Axonal guidance/chemorepulsive molecule		[188, 190]

Sema 3A	Localized to PNNs; resticition of plasticity through synaptic stabilization in mature neurons; localized to glial scar postinjury	Sema3A inhibitor (SM-216289)	[147, 189, 191–193]

Sema 3B	Localized to PNNs; restriction of plasticity through synaptic stabilization in mature neurons	Inhibitor not yet tested in CNS	[147]

Sema 4D	Localized to myelinating oligodendrocytes in injury	Inhibitor not yet tested in CNS	[194]

## Abbreviations

*α*9*β*1: Alpha9 beta1 integrin
AMPA: *α*-amino-3-hydroxy-5-methyl-4-isoxazolepropionic acid
BBB: Blood-brain barrier
Bral2/Hapln4: Brain-specific link protein 2
Ca^2+^: Calcium
ChABC: Chondroitinase ABC
CHO: Chinese hamster ovary cells
CNP: 2′, 3′-cyclic nucleotide phosphodiesterase
CNS: Central nervous system
CP: Critical period
Crtl1/Hapln1: Cartilage link protein 1
CSPGs: Chondroitin sulfate proteoglycans
CST: Corticospinal tract
DRG: Dorsal root ganglia (ganglion)
ECM: Extracellular matrix
FnD: Fibronectin III binding domain
GAG: Glycosaminoglycan side chains
HAS: Hyaluronan synthase
K^+^: Potassium
LARG: Leukemia-associated Rho guanine exchange factor
LINGO-1: Leucine-rich repeat and immunoglobin-like domain-containing protein-1
LTD: Long-term depression
LTP: Long-term potentiation
MAG: Myelin-associated glycoprotein
MAIs: Myelin-associated inhibitors
NG2: Neuron-glial antigen 2
NgR1: Nogo-66 receptor 1
NMDA: N-methyl-D-aspartate
NSPCs: Neural stem and progenitor cells
OMgp: Oligodendrocyte myelin glycoprotein
OPCs: Oligodendrocyte progenitor cells
p75NTR: p75 neurotrophin receptor
PirB: Paired immunoglobin-like receptor B
PLP/DM20: (Myelin specific) proteolipid protein
PNNs: Perineuronal nets
PNS: Peripheral nervous system
PTP*σ*: Protein tyrosine phosphatase sigma
RPTP*β*: Phosphacan/receptor-type-protein-tyrosine phosphatase *β*
SCI: Spinal cord injury
Sema3A: Semaphorin 3A
Sema4D: Semaphorin 4D
SIPR2: Sphingolipid 1-phosphate receptor 2
STAT3: Signal transducer and activator of transcription - 3
TK+GCV: Thymidine kinase + ganciclovir
TN-C: Tenascin C
TN-R: Tenascin R
TROY: Tumor necrosis factor receptor superfamily member.
